# Promoting inclusion in COVID-19 research for diverse Hispanic/Latino(x) populations: Recommendations from the RADx® Underserved Populations Hispanic/Latino/Latinx working group

**DOI:** 10.1017/cts.2024.600

**Published:** 2025-01-20

**Authors:** Rosa M. Gonzalez-Guarda, Edna Acosta-Perez, Cristina Adames, Rocio Bailey, Luis Carvajal-Carmona, Jenni Detwiler, Linda K. Ko, Hailey Leiva, Kathleen Page, Antoinette Schoenthaler

**Affiliations:** 1 Duke University School of Nursing, Durham, NC, USA; 2 Medical Science Campus, University of Puerto Rico, San Juan Puerto Rico, USA; 3 Hispanic Services Council, Tampa, FL, USA; 4 Department of Biochemistry and Molecular Medicine, University of California Davis, Sacramento, CA, USA; 5 University of North Carolina, Chapel Hill, NC, USA; 6 Department of Health Systems and Population Health, University of Washington, Seattle, WA, USA; 7 Johns Hopkins University School of Medicine, Baltimore, MD, USA; 8 New York University Grossman School of Medicine, New York, NY, USA

**Keywords:** Inclusion, diversity, Hispanic/Latino communities, minority populations, equity

## Introduction

Hispanic/Latino(x) communities, the largest US racial/ethnic minoritized population [1], experienced significant health inequities in COVID-19 morbidity and mortality [2,3], and had the largest decline in life expectancy [4]. These inequities are rooted in systemic racism that led to increased exposure to COVID-19 for this population (e.g., essential workers with crowded living arrangements), limited access to health care [5], and underenrollment in research [6]. Including Hispanic/Latino(x) populations in COVID-19 research is necessary to ensure scientific equity in benefits from public health advances.

The National Institutes of Health (NIH) created the RADx® Underserved Populations (RADx-UP) program to ensure that all Americans have access to COVID-19 testing, focusing on communities most affected by the pandemic. RADx-UP is a consortium of more than 137 research projects in communities across the United States (US), including all territories and Tribal Nations, coordinated by the Coordination and Data Collection Center (CDCC), which is led by the Duke Clinical Research Institute and the Center for Health Equity Research at UNC-Chapel Hill, in partnership with Community-Campus Partnerships for Health. Working Groups are a cornerstone to community engagement activities of the CDCC in which RADx-UP projects aim to address specific challenges and collaborate on resources and scholarship.

**Figure 1.**
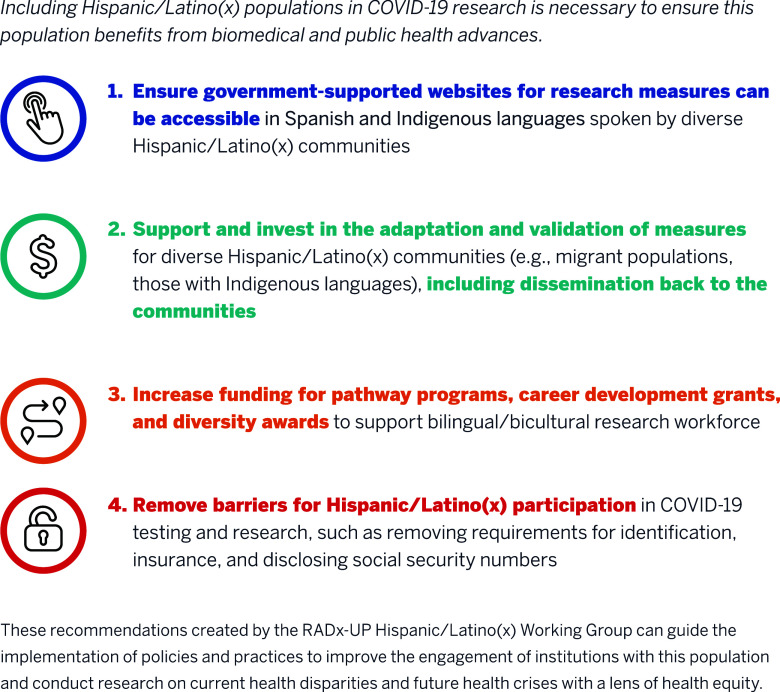
Promoting inclusion in COVID-19 research for diverse Hispanic/Latino(x) Populations: 4 recommendations.

This article summarizes recommendations made by the Engaging Hispanic/Latino/Latinx (HLL) Populations Working Group, which comprises community partners and academic researchers engaged in RADx-UP research, through a consensus-building process that occurred during monthly working group meetings. A list of recommendations from the HHL Working Group is presented below (see full list in Fig. 1) for decision-makers when designing research grants, funding opportunities, and resources for this population.

## Recommendation

### Ensure government-supported websites serving as a clearing house for research measures can be accessible in Spanish and Indigenous languages spoken by diverse Hispanic/Latino(x) community members

Although there has been an increased effort to build the capacity of community practitioners to find and use evidence-based practices [7] and measures have been developed in Spanish for COVID-19 research [8], most government websites supporting libraries of validated research measures are available only in English. For example, although the NIH has promoted the use of standard measures for COVID-19 research, such as those included in the PhenX Toolkit and the Disaster Research Response (DR2) Resource Portal, the websites providing these measures (https://www.phenxtoolkit.org/ and https://tools.niehs.nih.gov/dr2/, respectively) are in English only. To ensure diverse teams of researchers, government-supported websites need a Spanish interface. They also need to be searchable by language and accessible to community members who may be foreign-born Spanish speakers. Additionally, it is important to recognize other Indigenous languages spoken by Hispanic/Latino(x) populations living in the US. Collaboration with major search providers such as Google™ and Microsoft™ could lead to better search engines and increase visibility and user accessibility.

### Support and invest in the adaptation and validation of measures for diverse Hispanic/Latino(x) communities (e.g., migrant populations, those with Indigenous languages), including dissemination back to the communities

Translating research measures into Spanish (and other Indigenous languages) is critical but insufficient. The NIH advocates using standardized research instruments to compare data across studies. However, it is essential to recognize that these tools may need to be adapted and validated for use in different cultures or contexts. Hispanic/Latino(x) people in the US are a heterogeneous group with diverse racial and cultural backgrounds, national origins, socioeconomic position, and immigration status [9], but are often treated as a monolith. Adapting research measures to account for all these differences requires significant input from the community members and can be time- and resource-intensive. Federal investment would permit grantees the time and resources to better develop measures sensitive to Hispanic/Latino(x) communities. Funding opportunities such as center grants and Clinical Translational Science Awards should have a core dedicated to translating, adapting, and validating material and to providing technical assistance to researchers and community partners. Investment should also be made for the training and capacity-building of junior researchers and community partners on methodological aspects of translation, adaptation, and validation of research measures.

### Increase funding for pathway programs, career development grants, and diversity awards to support a bilingual/bicultural research workforce, prioritizing building community capacity

In academic medicine and across health care professions, structural racism represents the dominant system that systematically advantages White providers and disadvantages individuals of color – often restricting access to training, opportunities for mentorship, independent scholarship, and career advancement. For example, extensive research has shown that underrepresented minority (URM) trainees and faculty are more likely to experience inadequate mentorship, fewer opportunities for academic advancement, and higher demands (often referred to as the “minority tax”), and are less likely to receive equitable salaries for their rank and tenure [10]. Eliminating these institutional inequities is imperative to support the recruitment and continued success of URM trainees and faculty in academic health professions. A specific focus on a bilingual/bicultural research workforce should be integrated into steps that have been delineated for diversifying the Clinical and Translational Science Award programs [11,12] and academia more broadly.

### Remove barriers for Hispanic/Latino(x) participation in COVID-19 testing and research, particularly requirements for identification, insurance, and disclosing Social Security numbers that systematically leave out undocumented individuals and other communities who have been disproportionately affected by COVID-19 cases

Historic mistrust of public health and government systems often obstructs the Hispanic/Latino(x) community from engaging in activities deemed nonessential for their well-being. Although research can help reduce health inequities, community members who participate do not see the outcome of their efforts in tangible ways. The risks of participating in research, which may include side effects from clinical trials requiring treatments that participants cannot afford or the possibility of identifying undocumented individuals, are too high for community members when they lack quality health care, paid time off, or citizenship. Also, low levels of English proficiency and health literacy feed fear and mistrust because informed consent may be confusing. Regaining the trust of the Hispanic community with trusted messengers (faith leaders and community health workers) who understand cultural nuances and can communicate messages of hope may be needed to persuade community members to participate in research. When designing measures intended for Hispanic/Latino/Latinx communities, it is important to consider which personal identifiers are requested and why they are sought.

## Conclusion

The recommendations presented by the HLL Working Group aim to capitalize on the experience of the community and academic partners engaged in the RADx-UP initiative who worked with Hispanic/Latino(x) communities. These recommendations can guide the implementation of policies and practices that will improve engagement with this population when conducting research on pressing public health problems. Although made in the context of the COVID-19 pandemic, these recommendations extend far beyond the RADx-UP initiative and can help address current and future health crises through a lens of health equity.
